# Analysis of Patients with NET G1/G2 Neuroendocrine Tumors of the Small Intestine in the Course of Carcinoid Heart Disease—A Retrospective Study

**DOI:** 10.3390/jcm12030790

**Published:** 2023-01-19

**Authors:** Sonia J. Konsek-Komorowska, Mariola Pęczkowska, Agnieszka D. Kolasińska-Ćwikła, Andrzej Cichocki, Marek Konka, Katarzyna Roszkowska-Purska, Jarosław B. Ćwikła

**Affiliations:** 1 Department of Cardiology and Internal Medicine, School of Medicine, Collegium Medicum, University of Warmia and Mazury in Olsztyn, 10-082 Olsztyn, Poland; 2 The Cardinal Stefan Wyszyński National Institute of Cardiology, 04-628 Warsaw, Poland; 3 The Maria Sklodowska-Curie National Research Institute of Oncology, 02-034 Warsaw, Poland; 4 Diagnostic and Therapeutic Center–Gammed, 02-351 Warsaw, Poland

**Keywords:** carcinoid heart disease, carcinoid syndrome, neuroendocrine neoplasms of the small intestine, overall survival, primary tumor resection

## Abstract

Neuroendocrine neoplasms of the small intestine (SI-NENs) are one of the most commonly recognized gastroenteropancreatic neuroendocrine neoplasms (GEP-NENs). Carcinoid heart disease (CHD) is the primary cause of death in patients with the carcinoid syndrome (CS). The aim of this retrospective study was to evaluate possible factors impacting upon overall survival (OS) in subjects with both neuroendocrine tumors (NETs) G1/G2 of the small intestine (SI-NET) and CHD. Enrolled in our study of 275 patients with confirmed G1/G2 SI-NET, were 28 (10%) individuals with CHD. Overall survival was assessed using the Kaplan–Meier method. The Cox–Mantel test was used to determine how OS varied between groups. A Cox proportional hazards model was used to conduct univariate analyses of predictive factors for OS and estimate hazard ratios (HRs). Of the 28 individuals with confirmed carcinoid heart disease, 12 (43%) were found to have NET G1 and 16 (57%) were found to have NET G2. Univariate analysis revealed that subjects with CHD and without resection of the primary tumor had a lower OS. Our retrospective study observed that patients who presented with CHD and without resection of primary tumor had worse prognosis of survival. These results suggest that primary tumors may need to be removed when feasible, but further research is needed. However, no solid recommendations can be issued on the basis of our single retrospective study.

## 1. Introduction

Neuroendocrine neoplasms (NENs) are a relatively rare and heterogeneous class of tumors, originating from specialized neuroendocrine cells found throughout the body. Gastroenteropancreatic neuroendocrine neoplasms (GEP-NENs) account for 70% of all NENs, 2% of all gastrointestinal tract tumors [1,2,3,4,5].

Gastroenteropancreatic neuroendocrine tumors’ (GEP–NETs) incidence rates are progressively increasing globally [5]. The small intestine accounts for 37.4% of primary tumor cases [3]. In some studies, a slightly higher incidence of neuroendocrine neoplasms of the small intestine (SI-NENs) was observed in males (5.35/100,000/year) compared to females (4.76/100,000/year) [3,6].

From 2000, all GEP-NENs were recognized in accordance with the recommendations of the European Neuroendocrine Tumor Society (ENETS) based on the assessment of cell and organ site, histological type based on differentiation and histological maturity grading (G), pathomorphological advancement (pTNM), and clinical advancement staging [3,7,8,9]. Pathological classification of NENs is currently based on the recommendations of the World Health Organization (WHO) 2019 and of the American Joint Committee on Cancer/Union for International Cancer Control (AJCC/UICC) 2017, derived from the ENETS/WHO 2010 classification [3,10,11,12].

According to ENETS guidelines, all individuals with neuroendocrine neoplasms of the small intestine ought to be regarded as possible candidates for curative primary tumor resection (PTR) and resection of regional lymph node metastases [2].

Carcinoid syndrome (CS) is the most common paraneoplastic syndrome, comprising of the signs and clinical symptoms associated with hormonal activity of the SI-NENs. The typical form of CS involves exorbitant secretion of serotonin and/or other compounds with biological activity such as histamine, prostaglandins, kallikrein, and tachykinin [2,13,14].

Carcinoid heart disease (CHD) is the most serious complication of CS, which is marked by the deterioration of fibrotic valves, particularly in the right heart chambers. Isolated tricuspid valve (TV) regurgitation is present in up to 90% of individuals and primarily leads to a worsening of right ventricular function [15,16,17,18]. CHD is found in approximately 20–40% of cases of carcinoid syndrome [15,16].

The development of valvular CHD appears heavily dependent upon serotonin activity, and medical treatments focus on targeting the serotonin biochemical pathway [19].

The mainstay of imaging for carcinoid heart disease is transthoracic two-dimensional echocardiography (2D TTE). TV regurgitation in subjects with carcinoid heart disease is typically described by thickened TV leaflets, decreased mobility, and occasionally, immobility of the TV leaflets [20].

Individuals with cardiac involvement have a significantly worse long-term prognosis, with on average a 31 percent 3-year survival rate, which is half that of subjects without cardiac involvement [17,21,22,23]. Without therapy, the prognosis for valvular CHD is poor, with a median overall survival (OS) of only 11 months in individuals with progressive heart failure (HF) [19,24]. Therefore, identification of prognostic factors in the evolution of CHD is of fundamental clinical importance. Hence the aim of our study was to evaluate potential prognostic factors and the OS in subjects with well-differentiated G1 and G2 SI-NET exclusively in patients with diagnosed CHD.

## 2. Materials and Methods

The protocol for this retrospective study was accepted by the institution’s ethics committee (no. 18/2018), and informed consent was signed by all subjects. Patients diagnosed and treated between 2004 and 2019 with confirmed SI-NETs (i.e., the ileum and jejunum) were enrolled. Diagnosis of SI-NET was achieved with a multimodality approach including clinical and biochemical investigation comprising of chromogranin A or/and urinary 5-hydroxyindoleacetic acid (u5-HIAA), radiological and nuclear imaging techniques, or final surgery/histopathology reports where definitive surgery was undertaken. Histopathology reports included analysis of the primary tumor or metastases in the case of surgery with intention to treat (ITT). In the event of non-resectable lesions, tumor sampling was performed. In all patients a pathologist specialized in NET reported and verified the histology results. The histopathology reports included histological grade and the stage of the neuroendocrine neoplasm (pTNM) according to the WHO 2019 and AJCC/UICC 2017 classification [10]. u5-HIAA was measured at the first visit and then at follow-up or on suspicion of NET recurrence or progression.

The inclusion criteria for patients with CS and confirmed SI-NET were defined as typical clinical signs and symptoms of CS such as diarrhea, flushing of the skin, asthma-like symptoms (wheezing or/and shortness of breath), as well as elevated levels of biochemical markers such as chromogranin A and/or u5-HIAA.

Subjects with CS were screened for CHD on a regular basis using transthoracic echocardiography (TTE), if u5-HIAA and/or N-terminal pro–B-type natriuretic peptide (NT-proBNP) levels were markedly increased and/or presentation of signs and symptoms of heart disease occurred.

Patients with CHD were included either on the basis of signs and symptoms of heart disease or where typical cardiac involvement was identified using 2D TEE interpreted by a physician who was aware of anomalies caused by CHD. All individuals with thickened and retracted TV leaflets failing to coapt and diagnosed by Doppler echocardiography regurgitation moderate or severe were included.

In this retrospective study we primarily focused on patients with diagnosed CHD and analyzed potential prognostic factors and OS in this group of subjects.

In patients without information about the size of the tumors in histopathology reports, tumor size evaluation was based on structural imaging CT/MRI. We used the upper limit of normal to compare results of the concentration of u5-HIAA from different laboratories. OS was studied in all subjects with SI-NETs. The Kaplan–Meier method was used to assess OS. OS was calculated from the time of the initial diagnosis of SI-NET to either the date of death or the last follow-up visit. The Cox–Mantel test was used to evaluate the differences in OS between the groups. Univariate analyses were utilized to determine variables linked with overall survival and were performed utilizing a Cox proportional hazards model which estimated hazard ratios (HRs) and 95% confidence intervals (CIs). The primary and secondary efficacy endpoints of the retrospective study are summarized in Table 1.

All statistical calculations were performed utilizing Dell Inc. (2016), Dell Statistica (data analysis software system), version 13 (software.dell.com, accessed on 12 August 2022), with the level of statistical significance set at *p* < 0.05.

Histological and clinical data comprising evaluation of tumor type based on WHO 2019 and AJCC/UICC 2017 classification, including Ki-67 and the initial clinical stage of disease were accessible for analysis.

## 3. Results

A total of 275 patients with confirmed SI-NETs (i.e., the ileum and jejunum) were enrolled, including 142 females and 133 males (ratio 1.07:1). Patients and tumor characteristics are presented in Table 2.

**Figure 1:**
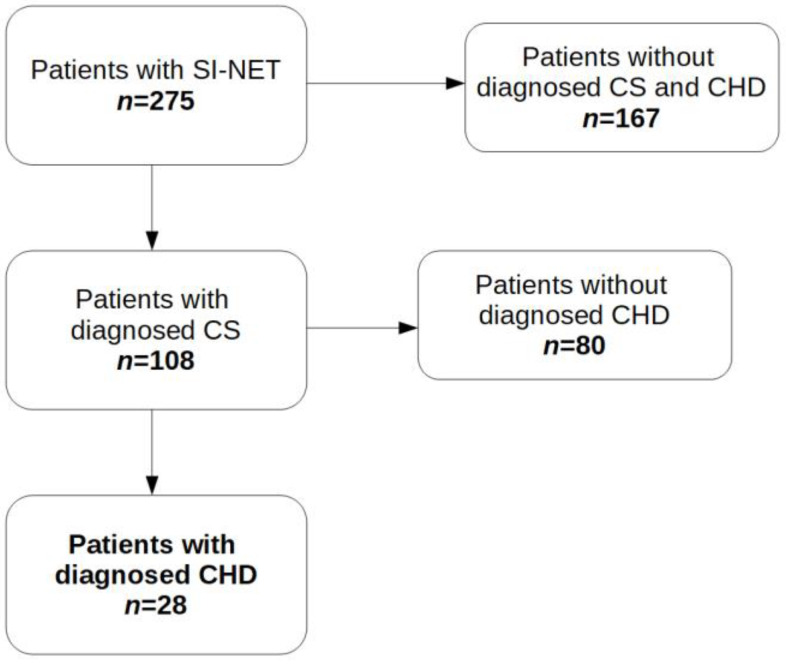
Patients enrolled in this retrospective study. Abbreviations: CHD, carcinoid heart disease; CS, carcinoid syndrome; SI-NET, neuroendocrine tumor of the small intestine.

There were 167 subjects without diagnosed CS and CHD and 108 patients with CS. Of these 108 patients, 28 with progression to CHD were evaluated (Figure 1).

Twelve subjects with NET G1 (43%) and 16 with NET G2 (57%) in the course of CHD were analyzed. Table 2 provides a summary of the clinical and pathological data that were taken into account in this study. This analysis revealed that pT3 and pT4 tumors were most frequently seen in subjects with NET G2. Distant metastasis (M) and clinical stage IV were noted in 96% of patients with CHD.

Arterial hypertension (68%), diabetes (14%), hyperlipidemia (14%), chronic kidney disease (14%), hypothyroidism (14%), atrial fibrillation (11%), and chronic obstructive pulmonary disease (7%) were the most common comorbidities found in patients with CHD.

There were six patients (32%) with confirmed synchronous CHD. The mean time between the initial diagnosis of SI-NET and the confirmation of CHD was 47.8 months in all patients, 64.8 months in patients that underwent primary tumor resection, and 28.4 months in subjects without primary tumor resection.

Over half of patients with CHD (54%) included in this study underwent primary small bowel tumor resection which was performed within three months from the initial diagnosis of SI-NET. Those subjects were considered for curative PTR and resection of regional lymph node metastasis at the initial diagnosis of SI-NET. In all patients with distant metastases, the decision of whether to conduct PTR was determined by analysis of comorbidities by a multidisciplinary team and also by a reasonably achieved curative approach including the curative resection of the distant metastases (generally liver metastases). None of the subjects needed palliative PTR due to symptoms related to small intestine obstruction, tumor bleeding, or (impeding) occlusion to avoid clinical deterioration or death.

Since 26 (93%) of the patients received somatostatin analogues (SSA), 18% of them did not have classic CS symptoms. However, diarrhea and flushing were still the most frequent symptoms present in subjects with CHD. Two patients refused treatment with somatostatin analogues despite clinical indications.

All patients had tricuspid valve regurgitation, either moderate (36%) or severe (64%) and characteristic in CHD valve leaflets anomalies seen in TTE (Table 3). A total of 54% of patients had pulmonary valve regurgitation, either mild (32%), moderate (7%), or severe (14%). Typical and prevalent symptoms of right-sided HF, such as shortness of breath, fatigue, and peripheral oedema, were present in patients with progressive CHD. 21% of subjects had tricuspid valve replacements, one had a concomitant pulmonary valve replacement, and one had a mitral valve replacement. The decision of whether to replace the valve was based on a multidisciplinary evaluation of general operability in regard to oncological status and cardiac function. Symptoms of heart failure improved in 66% patients that underwent cardiac surgery.

The median maximum concentration of u5-HIAA for patients with CHD (*n* = 26) was 31.5 times the upper limit of normal (ULN), and the median maximum concentration of NT-proBNP (*n* = 23) was 622.7 pg/mL (normal range: <125 pg/mL).

The median OS for all individuals with CHD (*n* = 28) was 53.8 months (CI −/+95% 47.7–91.2), and significantly different depending on resection of the primary tumor: 80.6 months (CI −/+95% 60.98–143.06) vs. 41.6 months (CI −/+95% 33.17–67.48) in those without resection (Table 4).

The median levels of maximum u5-HIAA for patients with CHD that underwent primary tumor resection were 8.05 ULN and 41.67 ULN in subjects without primary tumor resection.

**Figure 2:**
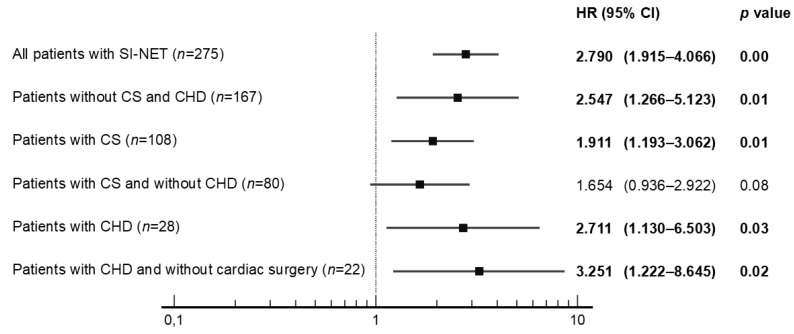
Univariate analysis of primary tumor resection (no vs. yes) with hazard ratios (HR) in all patients with SI-NET. Abbreviations: CHD, carcinoid heart disease; CS, carcinoid syndrome; SI-NET, neuroendocrine tumor of the small intestine.

**Figure 3:**
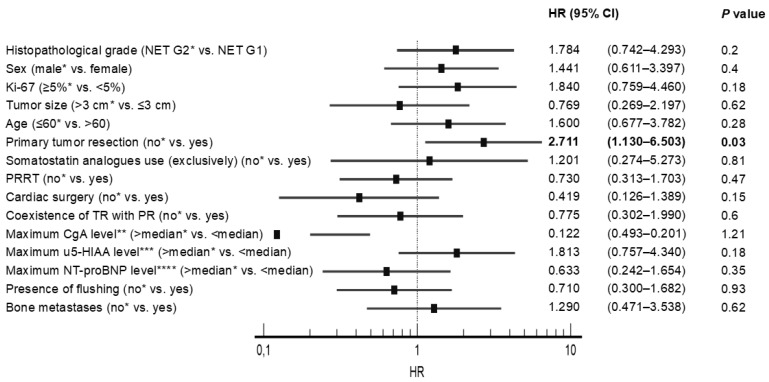
Univariate analysis with hazard ratios (HR) in patients with carcinoid heart disease. * Reference; ** Median maximum chromogranin A (CgA) level–17.1 ULN; *** Median maximum 5-hydroxyindoleacetic acid level in urine (u5-HIAA)–31.5 ULN; **** Median maximum N-terminal pro-B-type natriuretic peptide (NT-proBNP) level–622.7 pg/mL. Abbreviations: CgA, chromogranin A; NT-proBNP, N-terminal pro-B-type natriuretic peptide; PR, pulmonary valve regurgitation; PRRT, peptide-receptor radionuclide therapy; TR, tricuspid valve regurgitation; u5-HIAA, 5-hydroxyindoleacetic acid in urine.

Univariate analysis revealed that no PTR was significantly associated with poorer OS in all subjects with SI-NET G1/G2 (*n* = 275), in patients without CS and CHD (*n* = 167), in patients with CS (*n* = 108), and in patients with CHD (*n* = 28), with a Hazard Ratio (HR) presented in Figure 2 and Figure 3. On the contrary, other potential prognostic factors such as histopathological grade (G), gender, age, primary tumor size, Ki-67, cardiac surgery, stable disease due to somatostatin analogues use, peptide-receptor radionuclide therapy, coexistence of tricuspid regurgitation with pulmonary regurgitation and presence of bone metastasis were not significantly related to the OS in patients with CHD (Figure 3).

The most common cause of death throughout the follow-up period was progressive heart failure (*n* = 7) as well as tumor burden (*n* = 11) and gradual dysfunction of major organs (primarily the kidney and liver) (*n* = 5). Early mortality (within 30 days of admission) following cardiac surgery was noted in two patients: one patient died from cardiac failure during open heart surgery, and one died from heart failure progression during the perioperative period.

The median OS for all analyzed patients with SI-NET G1/G2 without CS and CHD (*n* = 167) was 169 months, and differed significantly depending on resection of the primary tumor: 170.2 months vs. 70.2 months in those without resection. The median OS for all patients with CS (*n* = 108) also differed significantly depending on resection of the primary tumor: 79.2 months vs. 53.9 months in those without resection. On the contrary, the median OS for all patients with CS and without CHD (*n* = 80) was 73 months, and did not differ significantly (*p* > 0.05) depending on resection of the primary tumor: 75.7 months vs. 70.2 months in those without resection.

## 4. Discussion

CHD is a serious complication of CS in individuals with SI-NETs [16] and is linked to increased morbidity and mortality, as well as associated with a general worsening of prognosis, when compared to patients who do not have CHD [25,26,27]. For this reason, clinical practice recommendations that are consistent and practical for CHD screening, diagnosis, and therapy are needed [28]. In this context, focusing on prognostic factors that influence overall survival (OS) in CHD appear to be beneficial.

Approximately 29% of all patients included in our retrospective analysis have carcinoid syndrome (without diagnosed carcinoid heart disease). CS progressed to CHD in approximately 26% of patients, and was diagnosed in around 10% of all subjects with SI-NETs.

The survival benefit of PTR in metastatic disease (resectable and unresectable) in all individuals with SI-NETs either with or without carcinoid heart disease has been well-documented [23] and can be linked to diminished synthesis of vasoactive substances [21] and a decrease of potential lethal consequences including intestinal obstruction due to tumor development and subsequent blockage [23].

It is widely recognized that subjects with SI-NETs and CS have a worse prognosis because of the risk of CHD development. To the best of our knowledge, our study is the first which may indicate that primary tumor resection is one of several factors improving OS in the selected group of patients with CHD. Univariate analysis indicated that no resection of primary tumor was significantly related to worse OS in patients with CHD, with a Hazard Ratio (HR) 2.71 (95% CI 1.13–6.50) and as well as in all subjects with G1/G2 SI-NETs (*n* = 275), in subjects without carcinoid syndrome and carcinoid heart disease (*n* = 167), and in individuals with carcinoid syndrome (*n* = 108). This research has practical significance in the context of an increasing trend to replace surgery with non-surgical therapies. On the contrary, other potential prognostic factors such as histopathological grade, gender, age, primary tumor size, Ki-67, cardiac surgery, stable disease due to peptide-receptor radionuclide therapy (PRRT), SSA use, coexistence of tricuspid regurgitation with pulmonary regurgitation and presence of bone metastasis were not significantly associated to OS. These results can be partially explained by the low number of individuals involved in our analysis, which increases the risk of type two errors. Consequently, based on our research, we cannot draw any firm conclusions on potential prognostic factors.

Uema et al. studied 139 subjects with carcinoid symptoms, advanced illness, and/or high urinary 5-hydroxyindoleacetic acid and discovered that removal of the primary tumor decreased mortality [28].

Significant results were recently published by Polcz et al. in a retrospective cohort analysis of 4076 subjects with metastatic neuroendocrine tumors of the small intestine, with 2025 (61.8%) undergoing PTR. Subjects who had PTR more often were younger, had been identified earlier, and had lower-grade SI-NET. The median OS was greater in PTR individuals compared to non-PTR subjects (71 vs. 29 months), *p* < 0.001 [29]. The results in our group of patients with CHD are therefore supported by this data. According to findings by Polcz et al., individuals treated at an academic or research institution were also less likely to have PTR, and PTR has decreased in prevalence with time. One explanation may be the growing usage of SSA in first-line therapy for advanced (metastatic) illness, which provide effective symptom relief and prevention of tumor progression. Though the outcomes suggest that these variables were also linked to the amended survival, it needs to be stated that these results relate to the whole group of subjects with neuroendocrine tumors of the small intestine—both with and without carcinoid heart disease—and despite confirmed benefits of progression-free survival, no overall survival benefit with SSA has been proved thus far [29,30]. Also implied is the tendency to choose other treatments instead of surgery, especially in elderly patients with many co-morbidities [29].

In our analysis, the mean time between the initial diagnosis of SI-NET and the diagnosis of CHD was 64.8 months in patients that underwent primary tumor resection, and 28.4 months in subjects without primary tumor resection. These results may show that primary tumor resection alone appears to provide an independent survival benefit in subjects with metastatic G1/G2 SI-NETs and CHD, and that it may be considered when feasible, to alleviate current symptoms and avoid future complications. However, due to the low number of patients and retrospective design of the study, no solid guidelines on resection of primary tumor in patients with CHD could be drawn from our analysis.

As was mentioned above in our CHD group, overall survival (OS) was significantly different depending on resection of the primary tumor: 80.6 months vs. 41.6 months in those without resection. These findings also demonstrate that, in the era of modern treatment approaches, overall survival increased, even in the group with initially worse prognoses. A median OS of 3.4 years has been revealed in subjects with carcinoid heart disease with a comparatively wide range in several publications (11 months without any treatment—6.5 years). It is also worth noting that prognosis is based on CHD severity [25,31]. When we consider that the scenario of valvular CHD without therapy is poor, with a median survival of only 11 months, we can see that the median OS has nearly quadrupled [19].

However, in our study on account of its retrospective design and immortal time bias due to different treatments over time, OS may not be the best outcome since it is mostly influenced by the burden of the tumor, particularly metastases, than other patient-centered outcomes, for example, symptoms or the necessity for additional surgical, radiologic, or endoscopic procedures.

A few analyses have consistently found a relationship between urinary 5-hydroxyindoleacetic acid levels, carcinoid heart disease diagnosis, and a negative influence on individual prognosis, indicating that urinary 5-hydroxyindoleacetic acid is a carcinoid heart disease predictor [26]. In our retrospective study, high concentration of u5-HIAA not significantly increased the risk of death. However, serotonin appears to be a fundamental factor in CHD development, as it promotes tissue fibrosis [32,33]. Furthermore, contrary to previous studies which support the use of measurements of u5-HIAA as a screening tool for CHD, our analysis showed that elevated u5-HIAA levels did not significantly correlate with survival in subjects with CHD, likely due to the low number of individuals included in the analysis [28,32,34,35].

Unfortunately, in contrast to reports indicating that valve surgery may increase survival in subjects with symptomatic severe right heart valve disease [36], our study showed no statistically significant difference in OS stratified by cardiac surgery in patients that undergo surgery or not. This can be as a result of low numbers of patients (21%) in our analyzed group that underwent cardiac surgery, despite around 64% presenting with severe TV dysfunction as identified by transthoracic echocardiography. Comparable research performed by Edwards et al. revealed that valve surgery did not enhance overall life expectancy from the time of the initial NET diagnosis [37].

There are several limitations of this study; the study was limited by its retrospective design. Although data on 275 patients was collected, this is likely not representative of the worldwide SI-NETs population, because of the lack of diversity in the patient cohort (White Caucasian). The low number of patients with CHD (*n* = 28) could confound the achieved results. We found that the paucity of detailed treatment factors in some patients, such as quality of surgery and systemic treatment, could confound the outcomes. There is a potential immortal time bias due to different treatments over time. The lack of knowledge of exact time of metastatic spread after primary tumor resection might have introduced bias.

## 5. Conclusions

Our retrospective study observed that no PTR was significantly associated with worse OS in patients with CHD. Unfortunately, no solid guidelines on PTR in patients with carcinoid heart disease could be drawn from our analysis. Our results may suggest that all subjects with SI-NETs, especially patients without PTR, require regular cyclic analysis of clinical status and TTE performance, but further studies, especially prospective clinical trials are needed to verify and confirm those initial achieved outcomes. However, due to the rarity of SI-NENs, it may be difficult to accomplish in the near future.

## Figures and Tables

**Table 1:** Summary of the primary and secondary efficacy endpoints for the patients with CHD. Abbreviations: CgA, chromogranin A; NT-proBNP, N-terminal pro-B-type natriuretic peptide; OS, overall survival; PR, pulmonary valve regurgitation; PRRT, peptide-receptor radionuclide therapy; TR, tricuspid valve regurgitation; u5-HIAA, 5-hydroxyindoleacetic acid in urine.

Endpoint	Definition/Measurement	Statistical Analysis
Primary efficacy		
Median OS	The time from the initial diagnosis of SI-NET to either the date of death or the last follow-up visit.	Kaplan–Meier method
Secondary efficacy		
Prognostic factors of OS	Histopathological grade (NET G2 vs. NET G1) Sex (male vs. female) Ki-67 (≥5% vs. <5%) Tumor size (>3 cm vs. ≤3 cm) Age (≤60 vs. >60) Primary tumor resection (no vs. yes) Somatostatin analogues (SSA) use (exclusively) (no vs. yes) PRRT (no vs. yes) Cardiac surgery (no vs. yes) Coexistence of TR with PR (no vs. yes) Maximum CgA level (>median vs. <median) Maximum u5-HIAA level (>median vs. <median) Maximum NT-proBNP level (>median vs. <median) Presence of flushing (no vs. yes) Bone metastases (no vs. yes)	Each factor was assessed for importance using univariate Cox proportional hazards model: factors were potentially associated with OS if the *p*-value was <0.05.

**Table 2:** Patients and tumor characteristics in neuroendocrine neoplasms of the small intestine.

	Carcinoid Heart Disease (CHD)			Carcinoid Syndrome (CS)	Patients without CHD and CS
	All	Primary Tumor Resection (PTR)	Without PTR		
	*n* = 28 (100%)	*n* = 15 (100%)	*n* = 13 (100%)	*n* = 80 (100%)	*n* = 167 (100%)
Female to male ratio	1.55	2.75	0.86	1.05	1.01
Mean age (range) in initial diagnosis	57.11 (36–76)	60.40 (46–76)	53.31 (36–72)	62.25 (34–84)	59.80 (20–87)
Size of the primary tumor (pathology) median, mm	22	20	25	29	22
Neuroendocrine tumor (NET) G1 (%)	12 (43)	8 (53)	4 (31)	31 (39)	116 (69)
Neuroendocrine tumor (NET) G2 (%)	16 (57)	7 (47)	9 (69)	49 (61)	51 (31)
Ki-67 (median)	3	2	3	3	2
pT (initial) - pT1 (%) - pT2 (%) - pT3 (%) - pT4 (%) - pTx or no data (%)	*n* = 28 0 (0) 3 (11) 12 (43) 4 (14) 9 (32)	*n* = 15 0 (0) 3 (20) 9 (60) 2 (13) 1 (7)	*n* = 13 0 (0) 0 (0) 3 (23) 2 (15) 8 (62)	*n* = 80 1 (12.5) 6 (7.5) 28 (35) 31 (39) 14 (17.5)	*n* = 167 16 (10) 38 (23) 66 (40) 32 (19) 15 (9)
N base on surgery/ follow-up/ imaging - N0 (%) - N1 (%) - No data (%)	*n* = 28 0 (0) 28 (100) 0 (0)	*n* = 15 0 (0) 15 (100) 0 (0)	*n* = 13 0 (0) 15 (100) 0 (0)	*n*= 80 3 (18) 76 (80) 1 (2)	*n* = 167 29 (17) 134 (80) 4 (2)
M base on surgery/follow-up/ imaging - M0 (%) - M1 (%) - No data (%)	*n* = 28 1 (4) 27 (96) 0 (0)	*n* = 15 1 (7) 14 (93) 0 (0)	*n* = 13 0 (0) 13 (100) 0 (0)	*n* = 80 6 (7.5) 74 (92.5) 0 (0)	*n* = 167 95 (57) 64 (38) 8 (5)
Clinical stage (initial) I–IIIa–local (%) IIIb–regional (%) IV–distal (%) No data (%)	*n* = 28 0 (0) 1 (4) 27 (96) 0 (0)	*n* = 15 0 (0) 1 (7) 14 (93) 0 (0)	*n* = 13 0 (0) 0 (0) 13 (100) 0 (0)	*n* = 80 1 (1) 5 (6) 74 (93) 0 (0)	*n* = 167 30 (18) 72 (43) 64 (38) 1 (1)
Primary tumor resection (PTR) (%)	15 (54)	15 (100)	0 (0)	51 (64)	146 (87)
Somatostatin analogues use (exclusively), stable disease (%)	26 (93)	14 (93)	12 (92)	68 (85)	78 (47)
Peptide–receptor radionuclide therapy (PRRT) (%)	14 (50)	10 (67)	4 (31)	41 (51)	13 (8)
Cardiac surgery (%)	6 (21)	3 (20)	3 (23)	0 (0)	0 (0)
5-hydroxyindoleacetic acid level in urine (%)	26 (93)	13 (87)	13 (100)	73 (91)	150 (90)
N-terminal pro-B-type natriuretic peptide (NT-proBNP) (%)	23 (82)	13 (87)	10 (77)	64 (80)	67 (40)
Transthoracic echocardiography (%)	28 (100)	15 (100)	13 (100)	76 (95)	30 (18)
Presence of flushing (%)	17 (61)	6 (40)	11 (85)	36 (45)	No data
Bone metastases (%)	8 (29)	3 (20)	5 (38)	No data	No data

**Table 3:** Patients with CHD–echocardiographic parameters.

	Carcinoid Heart Disease, *n* = 28 (100%)	Neuroendocrine Tumor (NET) G1, *n* = 12 (43%)	Neuroendocrine Tumor (NET) G2, *n* = 16 (57%)
Left ventricle (LV) internal dimension (diastolic dimension)–median, mm	4.2	4.7	4.2
Right ventricle (RV) diameter–median, mm	3.9	3.9	4.2
Right ventricular systolic pressure (RVSP)–median, mmHg	40	56	35
Left ventricular ejection fraction (LVEF)–median, %	65	60	65
Aortic diameter–median, (mm)	3.4	3.1	3.5
Mitral valve regurgitation (%) - Mild (%) - Moderate (%) - Severe (%)	19 (68) 16 (57) 2 (7) 1 (4)	7 (58) 5 (42) 1 (8) 1 (8)	12 (75) 11 (69) 1 (6) -
Tricuspid valve regurgitation (%) - Mild (%) - Moderate (%) - Severe (%)	28 (100) - 10 (36) 18 (64)	12 (100) - 5 (42) 7 (58)	16 (100) - 5 (31) 11 (69)
Aortic valve regurgitation (%) - Mild (%) - Moderate (%) - Severe (%)	9 (32) 9 (32) - -	3 (25) 3 (25) - -	6 (38) 6 (38) - -
Pulmonary valve regurgitation (%) - Mild (%) - Moderate (%) - Severe (%)	15 (54) 9 (32) 2 (7) 4 (14)	7 (58) 6 (50) - 1 (8)	8 (50) 3 (19) 2 (13) 3 (19)

**Table 4:** Median OS in patients with CHD.

Variable	Subjects	Overall Survival (Months)	95% Confidence Interval (CI)	*p* Value
Histopathological grade: - Neuroendocrine tumor G1 - Neuroendocrine tumor G2	12 16	75.62 51.51	56.25–112.26 36.07–92.94	0.19
Sex: - Female - Male	17 11	74.76 49.26	48.84–116.68 39.16–81.23	0.4
Age: - ≤60 - >60	16 12	53.71 71.33	36.63–96.67 55.58–107.07	0.28
Ki-67: - 1–5% - ≥5%	19 9	70.96 49.94	53.39–116.44 31.85–76.75	0.17
Tumor size: - ≤3 cm - >3 cm	17 7	51.70 82.99	45.64–111.39 72.43–99.52	0.62
Primary tumor resection: - No - Yes	13 15	41.63 80.59	33.17–67.48 60.98–143.06	0.02
Somatostatin analogues use (exclusively), stable disease (SD): - No - Yes	2 26	- 58.89	- 47.83–93.54	0.81
Peptide-receptor radionuclide therapy: - No - Yes	14 14	79.16 52.77	48.37–98.05 40.02–106.53	0.46
Cardiac surgery: - No - Yes	22 6	73.01 40.22	49.20–102.62 35.52–66.0	0.14
Coexistence of tricuspid regurgitation with pulmonary regurgitation (moderate or severe): - No - Yes	22 6	58.79 52.77	50.81–106.29 29.48–95.32	0.6
Maximum 5-hydroxyindoleacetic acid level in urine *: - <Median - >Median	13 13	57.72 51.55	49.78–141.20 37.73–73.31	0.18
Maximum N-terminal pro-B-type natriuretic peptide (NT-proBNP) level **: - <Median - >Median	11 12	50.63 74.67	40.24–85.08 49.01–132.27	0.35
Presence of flushing: - No - Yes	11 17	53.07 70.91	42.48–109.91 43.30–103.70	0.93
Bone metastases: - No - Yes	20 8	64.62 44.91	49.13–81.07 38.69–183.87	0.62

* Median–31.50 times the upper limit of normal; ** median–622.7 pg/mL; normal range: <125 pg/mL.

## Data Availability

The data presented in this study are available upon request from the corresponding authors.
